# The Relationship between Job Demands and Employees’ Counterproductive Work Behaviors: The Mediating Effect of Psychological Detachment and Job Anxiety

**DOI:** 10.3389/fpsyg.2017.01890

**Published:** 2017-10-30

**Authors:** Yang Chen, Shuang Li, Qing Xia, Chao He

**Affiliations:** School of Management, China University of Mining and Technology, Xuzhou, China

**Keywords:** job demands, psychological detachment, job anxiety, counterproductive work behaviors, stressor-detachment model

## Abstract

This study aims to explore the relation between job demands and counterproductive work behaviors (CWBs). A cross-sectional sample of 439 coal miners completed a self-report questionnaire that assessed their job demands, psychological detachment, job anxiety, and CWBs in a Chinese context. The conceptual model, based on the stressor-detachment model, was examined using structural equation modeling. The results suggest that psychological detachment mediates not only the relation between job demands and job anxiety but also that between job demands and CWBs. Furthermore, the relation between job demands and CWBs is sequentially mediated by psychological detachment and job anxiety. Our findings validate the effectiveness of the stressor-detachment model. Moreover, we demonstrate that the underlying mechanism of the relation between job demands and CWBs can be explained by psychological detachment and job anxiety.

## Introduction

China is the world’s largest producer and consumer of coal (International Energy Agency, 2000), and using coal as a main source energy will continue for the foreseeable future (Yan et al., 2011; Xue and Ren, 2012). Consequently, the development of China’s national economy is influenced by the operations of coal enterprises. It has been shown that the smooth running of coal enterprises depends mainly on the coal miners’ job performance (Wei et al., 2015). However, coal enterprises have encountered an unavoidable problem in their day-to-day operations: coal miners’ counterproductive work behaviors (CWBs) (Peters and Clingan, 1989; Cao and Yang, 2010; Li et al., 2013).

Counterproductive work behavior has been defined as any voluntary act that violates organizational norms and, thus, harms or is intended to harm an organization or its members (Robinson and Bennett, 1995; Spector and Fox, 2005a). Scholars use different names to describe these behaviors, such as organizational misbehavior (Vardi and Wiener, 1996), workplace aggression (Neuman and Baron, 2005), antisocial behavior (Robinson and O’Leary-Kelly, 1998), and workplace deviance (Robinson and Bennett, 1997). All of these behaviors are included in the concept of CWBs (Sulea et al., 2015), and these types of voluntary behaviors include such negative aspects as theft, sabotage, aggression, absenteeism, violence, insulting others, indifference, rudeness, and violating safety procedures (Spector, 2005; Hystad et al., 2014; Cohen, 2016). Considering the nature of these behaviors, CWBs can undoubtedly have significant negative effects on organizations and their members (Bowling and Eschleman, 2010; Rotundo and Spector, 2010). CWBs have been found to exist very widely in enterprises (Appelbaum et al., 2007; Penney et al., 2011), such as banks (Amazue et al., 2014; Nawawi et al., 2017) and hospitals (Grieco, 1987; Ahmed et al., 2013). The United States Chamber of Commerce reports that around three quarters of employees have committed theft at least once (Shulman, 2005). Moreover, CWBs negatively impact individuals, groups, and organizations (Alias et al., 2013). For example, unauthorized web surfing costs organizations an estimated £300 million per year in lost productivity (Taylor, 2007). Given these high costs, many researchers have devoted efforts to studying CWBs. The majority of previous CWB studies focused mainly on personality (O’Neill and Hastings, 2011), social culture (Zhang et al., 2015), and job stressors (Zhou et al., 2014). However, very few empirical studies have adopted the perspective of recovery.

Recovery is an effective way to avoid stress; it is usually defined as the process during which an individual’s functioning returns to its prestressor level and in which strain is reduced (Kinnunen et al., 2011). Through recovery, an individual’s resources are replenished (Meijman and Mulder, 1998; Trougakos and Hideg, 2009). However, if their recovery is incomplete, individuals must devote more effort to their work to ensure tasks are completed on time, which may, in turn, cause strain. If this situation continues, health problems and sickness absence will occur (Meijman and Mulder, 1998). As a main recovery strategy, psychological detachment could help to avoid further consuming employees’ resources and provides opportunities for their replenishment (Germeys and De Gieter, 2016).

Accordingly, Sonnentag and Fritz (2015) has proposed the stressor-detachment model to reveal the mechanism between stressor and strain reactions and subsequent performance. In the original stressor-detachment model, the effect of job stressors on strain is moderated and/or mediated by psychological detachment, and strain can lead to changes in job performance (Sonnentag, 2011; Sonnentag and Fritz, 2015). Although numerous studies have been conducted based on this model, most explore the connections between occupational stressors, psychological detachment, and strain reaction, such as fatigue (Sonnentag, 2011), well-being (Fritz et al., 2010b) and sleep (Sonnentag et al., 2008; Pereira and Elfering, 2014). Far less research has considered the connections between stressors, psychological detachment, negative affect, and job performance. Previous studies emphasize the moderating effect of psychological detachment, and most suggest that this is not significant (Safstrom and Hartig, 2013; DeArmond et al., 2014). To date, few studies have examined the mediating effect of psychological detachment (Germeys and De Gieter, 2016).

To fill this gap, the present study examines the mediating effect of psychological detachment on the relation between job stressor (i.e., job demands) and negative affect (i.e., job anxiety). Moreover, we also test their influence on subsequent performance (i.e., CWBs).

## Literature Review and Hypotheses

### Job Demands and Employees’ CWBs

Job demands refer to factors of a job that require continuous physical and/or psychological effort or skills and consume certain physiological and/or psychological costs (Bakker and Demerouti, 2007). Three job demands have received particular focus in prior research: high workload (Bakker et al., 2004, 2010; Jourdain and Chênevert, 2007; Petitta and Vecchione, 2011; Schmidt and Diestel, 2013; van Doorn and Hülsheger, 2015), time pressure (Rijk et al., 1998; van der Doef et al., 2000), and work-family conflict (Bakker et al., 2004, 2008; Demerouti et al., 2004; Jourdain and Chênevert, 2007). All three influence employee performance. Focusing specifically on the work characteristics of coal miners, such as three shifts per day, hard manual work, and no vacations or weekends, many will perceive the workload to be high; as coal miners lose wages if they complete tasks after the prescribed deadline, they are likely to feel time pressure; furthermore, with their workplace located in a remote region, and no vacations or weekends, coal miners may have little opportunity to fulfill their family responsibilities, and so may perceive that work interferes with their family life. Thus, we focus specifically on workload, time pressure, and work-family conflict.

As mentioned earlier, as job demands increase, workers’ physiological and/or psychological resources are increasingly drained. This will cause negative work outcomes if enough requisite resources of individuals are not available (Demerouti et al., 2001). Many empirical studies have confirmed the existence of this relationship: for example, Schaufeli et al.’s (2002) study suggests that job demands could cause job burnout. Ceschi et al. (2016) also found that job demands cause CWBs through emotional exhaustion. Under circumstances of high job demands, the constant consumption of employees’ resources leaves them unable to cope with these demands. Consequently, task difficulty increases and work enthusiasm declines, increasing the likelihood of absenteeism and absence (Hakanen et al., 2006; Xanthopoulou et al., 2007). For example, Chen and Spector’s (1992) study confirmed that job demands (e.g., role conflict, role ambiguity, workload, and interpersonal conflict) were positively related to employees’ CWBs (e.g., workplace aggressive behaviors, theft, and waste behaviors).

The conservation of resources (COR) theory posits that all individuals tend to gain and maintain valuable resources. When such resources are threatened, lost, or insufficiently returned to cover the resources invested (Hobfoll, 1989), individuals may withdraw their efforts to conserve resources (Halbesleben and Bowler, 2007; Ng and Feldman, 2012; Kiazad et al., 2014). In line with this, as high job demands constantly consume employees’ resources (Bakker and Demerouti, 2007), they will reduce efforts to restore loss or obtain new resources, which will cause CWBs, such as sabotage or absenteeism (Kanten and Ulker, 2013; Amazue et al., 2014). Therefore, we propose that:

H1: Job demands (workload, time pressure, and work-family conflict) are positively related to CWBs.

### Mediating Role of Job Anxiety

Job anxiety is an emotional response to uncertainty, vague fear, insecurity, and worry as regards one or several constituents of a particular job (Srivastava, 1985; Lazarus, 1991). Research shows that job stressors are a barrier to achieving individual goals (Hacker, 2003), making the individual prone to anxiety (Skinner and Brewer, 2002). As a form of job stressor, job demands are likely to provoke fear of failing to achieve organizational goals, therefore leading to anxiety. This is because high job demands require employees to devote more efforts to sustain an expected performance level (Hakanen et al., 2006). Under high job demands (e.g., workload, time pressure and work-family conflict), employees’ physical strength and energy are consumed (Demerouti et al., 2001), potentially causing them to feel that the job exceeds their capabilities (Hakanen et al., 2005), which could lead to low job control (Parker, 1998). Hence, as employees feel uncertain and worried over whether they can complete tasks and successfully achieve goals, anxiety eventually emerges. Accordingly, we hypothesize:

H2: Job demands (workload, time pressure, and work-family conflict) are positively related to job anxiety.

Previous research on CWBs has confirmed that job anxiety influences these behaviors. For example, based on a study of 288 patients with chronic mental disorders, Muschalla and Linden (2014) found that job anxiety causes employee absenteeism. Moreover, research suggests that individuals with high job anxiety always feel dissatisfaction with their job (Bücker et al., 2014), and employees may seek to reduce job dissatisfaction by engaging in CWBs (Greenidge et al., 2014; Zhang and Deng, 2016). In addition, job anxiety could lead to decreased conscientiousness (Bruce and Lynch, 2011), and low conscientiousness is a predictor of employees’ CWBs, such as absenteeism and dishonesty (Salgado et al., 2013). Therefore, we hypothesize:

H3: Job anxiety is positively related to employees’ CWBs.

The stressor-emotion model posits that emotions may mediate the relation between stressors and CWBs. When individuals are exposed to a job stressor, they may feel stress, which has the potential to induce a range of negative emotions. To reduce negative emotions, employees are more prone to engage in CWBs to counter job stressors (Spector and Fox, 2005b). In accordance with this view, job anxiety (a form of negative emotion) (Balducci et al., 2012) may mediate the relationship between job demands and CWBs. This is because job demands are the stressor (Bakker et al., 2004) which may hinder achievement of the individual goals (Hacker, 2003). Under the stress of job demands, employees fear about failure to achieve work goals, therefore leading to job anxiety (Skinner and Brewer, 2002), and employees may seek to reduce job anxiety by engaging in CWBs (Muschalla and Linden, 2014). Combining theoretical arguments with hypotheses H1 to H3, job anxiety may be assumed to play a mediator role in the relation between job demands and CWBs. Therefore, we hypothesize:

H4: Job anxiety mediates the relation between job demands and employees’ CWBs.

### Psychological Detachment as a Mediator

Psychological detachment refers to individuals not engaging in job-related activities and mentally disengaging themselves from job-related thoughts and worries during off-work time. In short, it is the experience of leaving one’s work behind during non-work time (Sonnentag and Fritz, 2007). Numerous studies have demonstrated that the factors related to work, especially its stressors, have a great effect on psychological detachment (Sonnentag et al., 2010; Kinnunen et al., 2011). For example, Safstrom and Hartig (2013) provided evidence for the existence of this relationship. In their study, taking 173 university students who participated in challenging programs of advanced professional studies, high job demands were found to hinder psychological detachment. High job demands (e.g., workload and time pressure) lead to low psychological detachment. On the one hand, this may be explained by high workload and time pressure causing prolonged activation, which, in turn, inhibits mental disconnect from work-related issues (Brosschot et al., 2006). On the other hand, employees may need to continue working during off-job time and/or might anticipate in the evening that their workload will also be high the following day, which makes psychological detachment more difficult (Sonnentag, 2012). Thus, we propose that:

H5: Job demands are negatively related to psychological detachment.

Psychological detachment might affect employees’ CWBs. This is because psychological detachment during off-work time could help employees to recover from job stressors and recover resources (e.g., energy and physical strength) (Fritz et al., 2010b). Moreover, according to COR theory, when an individual’s resources are replenished, psychological strain is reduced and he/she is less likely to engage in CWBs (Penney et al., 2011). In addition, it is helpful for employees to show work engagement, when they get the resources are needed at work (Kühnel et al., 2009). And work engagement has been found to be negatively related to employees’ CWBs (Ariani, 2013). Accordingly, we hypothesize:

H6: Psychological detachment is negatively related to employees’ CWBs.

According to the stressor-detachment model, psychological detachment mediates the relation between job stressors and strains. Stressors can elicit strain reactions, which include physiological responses, psychological reactions, and negative behaviors (e.g., argument with a co-worker). If employees detach from work during off-work time, the effects of job stressors on employees are greatly diminished (Sonnentag and Fritz, 2015). Given that high job demands lead to strain reactions from employees, such as CWBs, if they are able to psychologically detach from the job outside work, the demands no longer consume their resources, which would help to reduce CWBs (de Jonge and Peeters, 2009). Combining theoretical arguments with hypotheses H1, H5, and H6, psychological detachment can be assumed to play a mediator role in the relation between job demands and CWBs. Therefore, we hypothesize:

H7: The relation between job demands and employees’ CWBs is mediated by psychological detachment.

Some studies argue that psychological detachment is related to job anxiety (Moreno-Jiménez et al., 2012; Moreno-Jiménez and Herrer, 2013). This is because psychological detachment has been shown to reduce employees’ worry (Sonnentag and Fritz, 2015), which will, in turn, reduce the possibility of anxiety (Srivastava, 1985). In addition, it is easier for employees who are psychologically detached from work to shift from a work role into a non-work role during downtime (Ashforth et al., 2000), thus avoiding the job anxiety caused by role conflict (Gomez, 2014). Flaxman et al. (2012) confirmed that psychological detachment has a negative effect on job anxiety, based on a study of 77 academic employees in the United Kingdom. In addition, while job demands drain an individual’s resources (e.g., energy and physical strength), psychological detachment could remove the negative effects of job demands on the individual. Removing the mental influence of job demands from the individual can enable resources depleted during work to be rebuilt (Kühnel et al., 2009), which may improve job control and, consequently, decrease job anxiety (Battams et al., 2014). Combining theoretical arguments with hypotheses H2 and H5, we hypothesize:

H8: Psychological detachment is negatively related to job anxiety.
H9: Psychological detachment mediates the relation between job demands and job anxiety.

**FIGURE 1:**
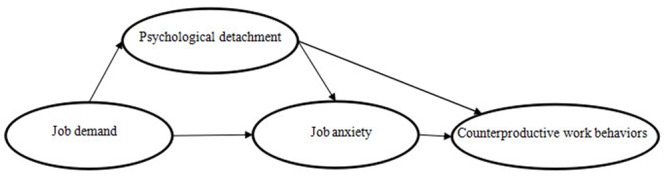
Hypothetical model (based on the stressor-detachment model).

Based on the stressor-detachment model and previous research, we develop a multivariate model to examine our hypotheses, especially the proposed mediating effect of psychological detachment and job anxiety on the relation between job demands and CWBs. The conceptual model we propose in the present study is depicted in **Figure 1**.

## Materials and Methods

### Participants and Procedures

#### Participants

The study’s participants are full-time coal miners from three large state-owned coal mines in northern China. The data were collected via paper-and-pencil questionnaires. In total, 540 questionnaires were issued and 487 were returned (90.2% response rate). Questionnaires less than 50% completed and those that contained illegible responses were removed (48 responses). Following these exclusions, 439 valid questionnaires remained (81.3% valid response rate).

Due to the nature and uniqueness of the industry, most coal company workers are men; thus, 90% of the valid respondents were men. The age groups were as follows: 12.3% aged under 25, 45.1% aged 26–35, 26.9% aged 36–45, 15.3% aged 46–55 years, and 0.5% aged 56 and above. Most of the participants have senior middle school education (33.1%), followed by junior college (22.8%), junior middle school (21.2%), bachelor degree and above (19.6%), and primary school and below (3.4%). On job tenure, 9.6% have worked for their organization for less than 1 year, 9.6% for 1–3 years, 13.4% for 3–5 years, 29% for 5–10 years, and 38.5% for more than 10 years. The respondents’ reported positions were as follows: general workers (82.1%), first-line managers (14.6%), middle managers (2.7%), and senior managers (0.7%). Most respondents were married (87.7%), while 10.9% were unmarried (10.9%) and 1.4% reported “other.”

#### Procedures

The data were collected over approximately 1 month from May to June 2017. First, we contacted each company’s HR manager to invite participation in our investigation; all of those we contacted were willing to allow their employees to participate. We then divided our research team into three groups, each of which was assigned to a different company. Assisted by the HR managers, 180 questionnaires were sent to workers at each of the three companies. Each questionnaire was accompanied by a cover letter explaining the purpose of the study, that participation was voluntary, and that participants’ privacy would be strictly protected. Invitees were required to complete the paper-and-pencil questionnaire within 20 min in a designated meeting room during meeting time. All the questionnaires were collected *in situ* when the 20 min elapsed. To convey our appreciation, participants were offered the gift of a high-quality pen for completing the questionnaire. This study was part of a larger research project on coal miners’ behaviors. Our study was approved by our university’s ethics committee.

### Measures

Each variable in the self-administered survey was measured using a multi-item scale, each of which was adopted from relevant prior research. As all our participants were Chinese, we followed the double-blind back-translation procedure (Schaffer and Riordan, 2003) to translate all items into Chinese. To avoid translation ambiguity, each item was translated by professional translators. The internal consistency of each scale was verified through Cronbach’s alpha.

#### Job Demands

##### Workload

This was assessed using the four-item scale of De Grood (2009), which was originally developed by Caplan et al. (1975). Participants’ responses were measured on a 5-point Likert scale, ranging from strongly disagree (1) to strongly agree (5). Sample items of this scale include, “My workload is too heavy in my job” and “I have to work very quickly to get everything done in my job.” The scale’s Cronbach’s alpha for this sample was 0.78.

##### Time pressure

This was assessed using a five-item scale adopted from Aleksić et al. (2017). Responses were measured on a 5-point Likert scale, ranging from strongly disagree (1) to strongly agree (5). The sample items include: “When working, I have only a limited amount of time to finish my work” and “When working, I am in a hurry.” The scale’s Cronbach’s alpha for this sample was 0.79.

##### Work-family conflict

This was assessed using a five-item scale developed by Netemeyer et al. (1996), designed to measure the extent to which employees feel that work interferes with their family life. Given the evidence that work interferes with family life to a greater degree than family life interferes with work (Frone et al., 1992; Eagle et al., 1997; Swanson et al., 1998; Emslie et al., 2004). Thus, in this study, we have chosen to focus on work-to-family conflict (which we refer to as ‘work-family conflict’). Responses were measured on a 5-point Likert scale, ranging from strongly disagree (1) to strongly agree (5). The sample items include: “The demands of my work interfere with my home and family life” and “The amount of time my job takes up makes it difficult for me to fulfill my family responsibilities.” The scale’s Cronbach’s alpha for this sample is 0.86.

#### Psychological Detachment

The four-item scale to measure psychological detachment was adopted from the work of Sonnentag and Fritz (2007). This scale refers to employees’ views on their non-work time over the past few weeks. Every item was rated on a 5-point Likert scale, ranging from fully disagree (1) to fully agree (5). Sample items include: “I don’t think about work at all during non-work time” and “I get a break from the demands of work during non-work time.” Cronbach’s alpha was 0.80.

#### Job Anxiety

The eight-item job anxiety scale developed by McCarthy et al. (2016) was applied to assess participants’ current job-related anxiety. Each item was measured on a 5-point Likert scale, ranging from absolutely disagree (1) to absolutely agree (5). Sample items include: “I am overwhelmed by thoughts of doing poorly at work” and “I often feel anxious that I will not be able to perform my job duties in the allotted time.” Cronbach’ s alpha was 0.87.

#### Counterproductive Work Behaviors

We assessed CWBs with a 19-item measure of quantitative CWBs developed by Robinson and Bennett (1995). Participants responded to each item on a 5-point Likert scale (1 = Never, 5 = Everyday). The scale is a multidimensional construct including two portions: (a) 7 items for CWB aims to the individual members (CWB-I) (α = 0.78), (b) 12 items for CWB aims to the organization (CWB-O) (α = 0.75). Examples of the statements include: “Made fun of someone at work” and “Dragged out work in order to get overtime.” Cronbach’s alpha of the whole scale was 0.73.

To rule out the potential confounding effects of demographic variables and some work-related background variables, we controlled for gender, age, education, job tenure, position, and marital status. First, gender was divided into two categories (0 = male; 1 = female). Second, to protect privacy, participants were divided into five age groups (1 = under 25; 2 = 26–35 years old; 3 = 36–45; 4 = 46–55; 5 = 56 and above). Third, education was divided into five categories (1 = primary school and below; 2 = junior middle school; 3 = senior middle school; 4 = junior college; 5 = bachelor degree and above). Job tenure was also divided into five categories (1 = under 1 year; 2 = 1–3 years; 3 = 3–5 years; 4 = 5–10 years; 5 = over 10 years). Fourth, to avoid the potential effects of position, we treated it as control variable by dividing into four categories (1 = general worker; 2 = first-line manager; 3 = middle manager; 4 = senior manager). Finally, marital status was divided into unmarried (1), married (2), and others (3).

### Data Analytic Strategy

For data analysis, SPSS version 21.0 was used to analyze the internal consistency, descriptive statistics, and correlations among the variables. In conducting the two-step approach to test the mediating effects, as suggested by Anderson and Gerbing (1988), we utilized AMOS version 22.0. First, the measurement model was tested using confirmatory factor analysis (CFA) to assess the variables’ discriminate validity (Cheung and Wong, 2011; Choi and Moon, 2017). The fit indices of the hypothesized factor model were compared with those of alternative factor models to confirm which better fit the data (Mathieu and Farr, 1991; Cheung and Wong, 2011). The second step, viable only after validation through the first step, was to use maximum likelihood structural equation modeling (SEM) to examine the structural relationships among the study variables.

The following indices were used to study the adequacy of the estimated model: goodness-of-fit index (GFI), χ^2^/df, normed fit index (NFI), root mean square error of approximation (RMSEA), and comparative fit index (CFI). An acceptable χ^2^/df is between 1 and 5 (Salisbury et al., 2002). The GFI, NFI, and CFI should each be over 0.90 (Salisbury et al., 2002). Finally, the RMSEA should be less than 0.08 (Byrne, 2006).

## Results

### Common Method Bias

Common method bias (CMB) can inflate relationships when the data are collected from a single source (Podsakoff et al., 2003). To determine whether CMB is problematic in this study, the CFA marker technique was employed (Podsakoff et al., 2012). We built the CFA (5-factor) model by adding the CMB variable to the 4-factor model. Compared with the 4-factor model (χ^2^/df = 4.29, GFI = 0.98, NFI = 0.95, CFI = 0.96, RMSEA = 0.068), the CFA (5-factor) model (χ^2^/df = 7.20, GFI = 0.93, NFI = 0.86, CFI = 0.87, RMSEA = 0.119) is no better. Further, the chi-square difference also did not reach the significant level [Δχ^2^(9) = 7.88, *p* > 0.05]. Thus, CMB was negligible in our study (see **Table 3**).

### Description

Before testing our hypotheses, we examined the intraclass correlation coefficient (ICC) (see **Table 1**). The result show that the data’s reliability is good.

**Table 1:** The intraclass correlation coefficient of the variables.

Name	Estimate value	95%CI	
		Lower bounds	Upper bounds
ICC1	0.852	0.829	0.872
ICC2	0.793	0.758	0.824
ICC3	0.844	0.820	0.866
ICC4	0.900	0.886	0.914

*ICC1, the intraclass correlation coefficient of job demands; ICC2, the intraclass correlation coefficient of psychological detachment; ICC3, the intraclass correlation coefficient of job anxiety; ICC4, the intraclass correlation coefficient of CWB.*

The means and standard deviations of and the correlations between each of the variables are presented in **Table 2**. Workload, time pressure, and work-family conflict are all positively related to employees’ CWBs (*r* = 0.25, *p* < 0.001; *r* = 0.21, *p* < 0.001; *r* = 0.46, *p* < 0.001) and job anxiety (*r* = 0.31, *p* < 0.001; *r* = 0.25, *p* < 0.001; *r* = 0.42, *p* < 0.001). Workload, time pressure, and work-family conflict were all significantly and negatively correlated with psychological detachment (*r* = -0.29, *p* < 0.001; *r* = -0.31, *p* < 0.001; *r* = -0.19, *p* < 0.001). Thus H1, H2, and H5 were each preliminary supported. Psychological detachment was negatively associated with CWBs (*r* = -0.21, *p* < 0.001) and job anxiety (*r* = -0.34, *p* < 0.001), providing preliminary support for H6 and H8. Job anxiety was related positively to CWBs (*r* = 0.47, *p* < 0.001), providing preliminary support for H3. As the correlations between the control variables and our study variables were either weak or not significant, we do not further consider the effects of control variables in subsequent analysis.

**Table 2:** Descriptive statistics and correlations among all variables.

	*M*	*SD*	1	2	3	4	5	6	7	8	9	10	11	12
(1) Gender	0.10	0.30	–											
(2) Age	2.47	0.91	–0.28***	–										
(3) Education	3.35	1.12	0.23***	–0.37***	–									
(4) Job tenure	3.76	1.31	–0.38***	0.57***	–0.31***	–								
(5) Position	1.23	0.52	0.00	0.02	0.29***	0.00	–							
(6) Marital status	1.90	0.34	–0.04	0.17***	0.01	0.21***	–0.03	–						
(7) Time pressure	2.93	0.97	–0.04	0.11*	–0.06	0.06	0.07	0.04	(0.79)					
(8)Workload	3.14	0.80	–0.02	0.04	0.05	0.06	0.05	0.02	0.50***	(0.78)				
(9) Work-family conflict	3.11	0.64	0.01	0.02	–0.07	–0.06	0.06	–0.01	0.24***	0.30***	(0.86)			
(10) Psychological detachment	2.82	0.81	–0.07	–0.10*	0.05	–0.02	0.03	–0.05	–0.31***	–0.29***	–0.19***	(0.80)		
(11) CWB	3.17	0.64	0.08	0.05	–0.02	–0.02	0.09	–0.02	0.21***	0.25***	0.46***	–0.21***	(0.73)	
(12) Job anxiety	3.39	0.69	–0.05	0.14**	0.00	0.02	0.11*	0.01	0.25***	0.31***	0.42***	–0.34***	0.47***	(0.87)

*Gender: (0 = male and 1 = female); age: (1 = under age 25, 2 = 26–35 year-old, 3 = 36–45 year-old, 4 = 46–55 year-old, 5 = 56 year-old and above); education: (1 = primary school and below, 2 = junior middle school, 3 = senior middle school, 4 = junior college, 5 = bachelor degree and above); job tenure: (1 = under 1 year, 2 = 1–3 years, 3 = 3–5 years, 4 = 5–10 years, 5 = above 10 years); position: (1 = general worker, 2 = first-line manager, 3 = middle manager, 4 = senior manager); marital status: (1 = unmarried, 2 = married, 3 = others). Cronbach’s alpha are in parentheses on the diagonal. ^∗^*p* < 0.05, ^∗∗^*p* < 0.01, ^∗∗∗^*p* < 0.001.*

### Measurement Model Testing

We next performed CFA using AMOS 22.0. To increase the accuracy of parameter estimates, the method of item parceling should be adopted to represent variables’ indicators (Rogers and Schmitt, 2004). Therefore, four latent factors (job demands, psychological detachment, job anxiety, and CWBs) and nine observed indicators were included in the study. When comparing with item-level data, the advantages of aggregate-level data (e.g., higher communality and lower random error) are obvious (Li et al., 2017). The measurement model was tested by comparing the fit indices between the single-factor model (job demands, job anxiety, psychological detachment, and CWBs combined into one factor), 2-factor model (job demands, job anxiety, and CWBs on the same factor; psychological detachment on the other), 3-factor model (job demands and job anxiety on the same factor; psychological detachment and CWBs as separate factors), and 4-factor model (job demands, job anxiety, psychological detachment, and CWBs as separate factors). The results suggested that the 4-factor model (χ^2^/df = 4.29, GFI = 0.98, NFI = 0.95, CFI = 0.96, RMSEA = 0.068) provided a better fit than the other models (see **Table 3**). In organizational behavior studies, the method explained above has been widely used in prior research (Mathieu and Farr, 1991; Cheung and Wong, 2011).

**Table 3:** Comparison of measurement model.

Structure	χ^2^	df	χ^2^/df	GFI	NFI	CFI	RMSEA
1- factor	372.23	27	13.79	0.85	0.65	0.67	0.171
2- factor	213.23	26	8.20	0.90	0.80	0.82	0.128
3- factor	188.61	24	7.86	0.92	0.82	0.84	0.125
4- factor	94.29	21	4.29	0.98	0.95	0.96	0.068
5- factor	86.41	12	7.20	0.93	0.86	0.87	0.119

*1-factor: JD (TP; WL; WC)+PD+JA+CWB; 2-factor: JD+JA+CWB; PD; 3-factor: JD, PD; JA+CWB; 4-factor: JD, PD, JA, CWB; 5-factor: JD, PD, JA, CWB, CMB. JD, job demands; TP, time pressure; WL, workload; WC, work-family conflict; PD, psychological detachment; JA, job anxiety; CWB, counterproductive work behavior; CMB, common method bias.*

### Structure Model Testing

**FIGURE 2:**
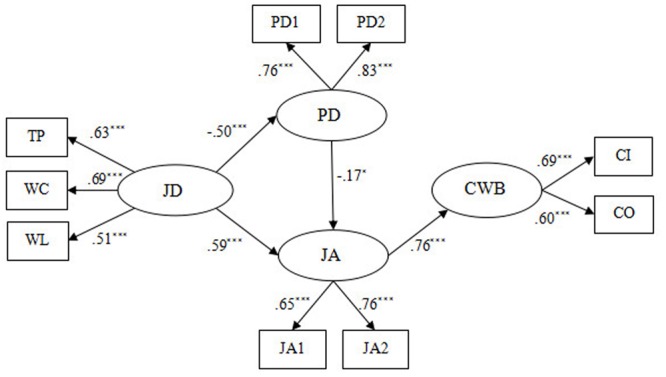
The original stressor-detachment model (Model 1). JD, job demands; TP, time pressure; WL, workload; WC, work-family conflict; PD, psychological detachment; JA, job anxiety; CWB, counterproductive work behavior; CI, CWB-I; CO, CWB-O; PD1 and PD2 aggregate of two items from Psychological Detachment Questionnaire, respectively; JA1 and JA2 aggregate of four items from Job Anxiety Questionnaire, respectively ^∗^*p* < 0.05, ^∗∗^*p* < 0.01, ^∗∗∗^*p* < 0.001.

We use SEM to examine the mediating effects of psychological detachment and job anxiety and to assess our proposed model. In the original stressor-detachment model, there is no direct effect from psychological detachment to subsequent performance. Thus, we built an alternative Model 1 (see **Figure 2**), in which the direct path from psychological detachment to CWBs was deleted from our Hypothetical model (based on the stressor-detachment model) (**Figure 1**). The results showed that Model 1 (χ^2^/df = 7.47, GFI = 0.92, NFI = 0.84, CFI = 0.86, RMSEA = 0.122) did not fit the data well. Compared with the Hypothetical model (χ^2^/df = 7.72, GFI = 0.92, NFI = 0.94, CFI = 0.86, RMSEA = 0.124), the chi-square difference did not reach the significant level [Δχ^2^(1) = 2.03, *p* > 0.05], suggesting that Model 1 did not fit the data better than the Hypothetical model (see **Table 4**).

**Table 4:** Comparison of the structural models.

Model	χ^2^	*df*	χ^2^/df	GFI	NFI	CFI	RMSEA
Hypothetical model	169.82	22	7.72	0.92	0.94	0.86	0.124
M1	171.85	23	7.47	0.92	0.84	0.86	0.122
M2	160.12	22	7.28	0.92	0.85	0.87	0.120
M3	83.16	21	3.96	0.97	0.94	0.95	0.073
M4	150.90	21	7.19	0.93	0.86	0.87	0.119

**FIGURE 3:**
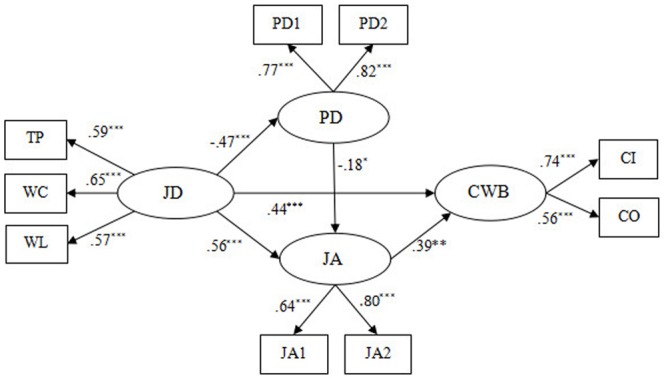
The alternative model bases on the Model 1 (Model 2). JD, job demands; TP, time pressure; WL, workload; WC, work-family conflict; PD, psychological detachment; JA, job anxiety; CWB, counterproductive work behavior; CI, CWB-I; CO, CWB-O; PD1 and PD2 aggregate of two items from Psychological Detachment Questionnaire, respectively; JA1 and JA2 aggregate of four items from Job Anxiety Questionnaire, respectively ^∗^*p* < 0.05, ^∗∗^*p* < 0.01, ^∗∗∗^*p* < 0.001.

We then built Model 2 (see **Figure 3**), in which a direct path from job demands to CWBs was added to Model 1. The results demonstrated that Model 2 (χ^2^/df = 7.28, GFI = 0.92, NFI = 0.85, CFI = 0.87, RMSEA = 0.120) also has unsatisfactory data fitting (see **Table 4**). Compared with Model 1, the chi-square difference [Δχ^2^(1) = 11.73, *p* < 0.05] reached a significant level, meaning that Model 2 is superior to Model 1.

**FIGURE 4:**
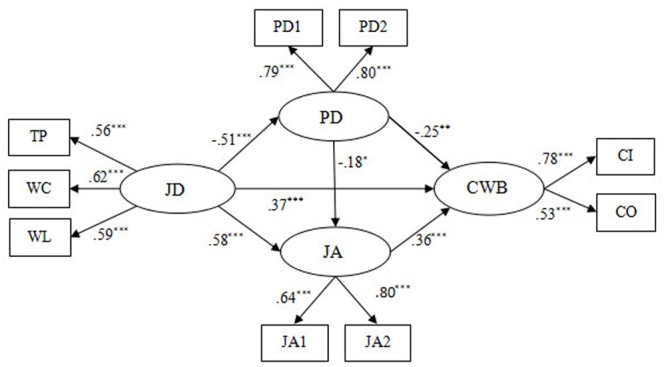
The ultimate mediation model (Model 3). *N* = 439. JD, job demands; TP, time pressure; WL, workload; WC, work-family conflict; PD, psychological detachment; JA, job anxiety; CWB, counterproductive work behavior; CI, CWB-I; CO, CWB-O; PD1 and PD2 aggregate of two items from Psychological Detachment Questionnaire, respectively; JA1 and JA2 aggregate of four items from Job Anxiety Questionnaire, respectively ^∗^*p* < 0.05, ^∗∗^*p* < 0.01, ^∗∗∗^*p* < 0.001.

To find the most satisfactory model, we then developed another alternative model (Model 3), in which a path from psychological detachment to CWBs was added to Model 2 (see **Figure 4**). The results demonstrated that Model 3 (χ^2^/df = 3.96, GFI = 0.97, NFI = 0.94, CFI = 0.95, RMSEA = 0.073) fitted the data well. Moreover, the factor loading of each indicator was between 0.53 and 0.80, and all reached the significant level (*p* < 0.001) (see **Figure 4**). Hence, all of the indicators are suitable to represent their latent constructs. Through comparison of the chi-square change between Model 3 and the Hypothetical model [Δχ^2^(1) = 86.66, *p* < 0.001], Model 3 and Model 1 [Δχ^2^(2) = 88.69, *p* < 0.001], and Model 3 and Model 2 [Δχ^2^(1) = 76.96, *p* < 0.001], the significant level was reached, revealing that Model 3 significantly improves model fit and is superior to all of the alternative models (see **Table 4**). Thus, Model 3 was selected as our study’s structural model (see **Figure 4**).

**FIGURE 5:**
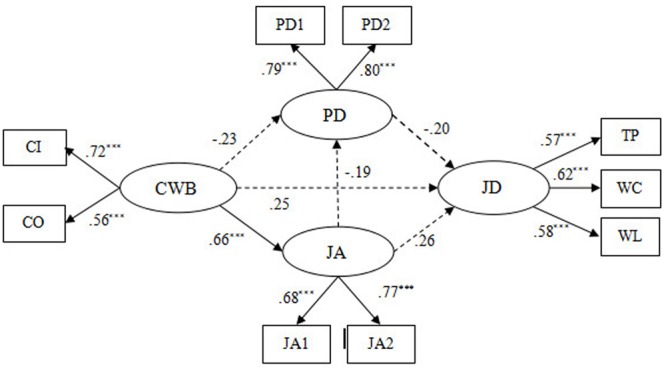
The reversed model (Model 4). JD, job demands; TP, time pressure; WL, workload; WC, work-family conflict; PD, psychological detachment; JA, job anxiety; CWB, counterproductive work behavior; CI, CWB-I; CO, CWB-O; PD1 and PD2 aggregate of two items from Psychological Detachment Questionnaire, respectively; JA1 and JA2 aggregate of four items from Job Anxiety Questionnaire, respectively ^∗^*p* < 0.05, ^∗∗^*p* < 0.01, ^∗∗∗^*p* < 0.001.

To determine the casual relationships between our study variables, we also test the reverse model (Model 4) (see **Figure 5**). The results show that the path coefficient from CWBs to job anxiety (β = 0.66, *p* < 0.001) is significant. However, the fit indices of the reverse model are unsatisfactory (see **Table 4**). Therefore, the reverse model is not acceptable.

Furthermore, the bootstrapping method was used to test the mediation effects in Model 3. Bootstrapping is the ideal way to test indirect effects as it does not impose distributional assumptions (Zhang et al., 2015). The most accurate estimation of indirect effects can be obtained by bootstrap sampling. If zero is not included in the 95% confidence interval, then indirect effects are significant. The results showed that our hypotheses are all verified (see **Tables 5, 6** and **Figure 4**). First, the total effect from job demands to CWBs was significant (β = 0.74, *p* < 0.001), supporting H1. Second, the total effect from job demands to job anxiety was also significant (β = 0.67, *p* < 0.01), supporting H2. Third, the direct effect from job anxiety to CWBs was significant (β = 0.36, *p* < 0.001), supporting H3. Fourth, the indirect effect of job demands on CWBs through job anxiety was significant (β = 0.21, *p* < 0.05), supporting H4. Fifth, the direct effect from job demands to psychological detachment was significant (β = -0.51, *p* < 0.001), supporting H5. Sixth, the direct effect from psychological detachment to CWBs reached the significant level (β = -0.25, *p* < 0.01), supporting H6. Seventh, the indirect effect of job demands on CWB via psychological detachment was significant (β = 0.13, *p* < 0.05), supporting H7. Eighth, the path coefficient between psychological detachment and job anxiety was significant (β = -0.18, *p* < 0.05), supporting H8. Ninth, job demands affected job anxiety through psychological detachment (β = 0.09, *p* < 0.05), supporting H9. Finally, we also demonstrated that the relation between job demands and CWBs was sequentially mediated by psychological detachment and job anxiety (β = 0.03, *p* < 0.05).

**Table 5:** The results of the study.

Hypothesis	Estimate effect	Get supported or not
H1	0.74***	Yes
H2	0.67***	Yes
H3	0.36***	Yes
H4	0.21*	Yes
H5	–0.51***	Yes
H6	–0.25**	Yes
H7	0.13*	Yes
H8	–0.18*	Yes
H9	0.09*	Yes

*^∗^*p* < 0.05, ^∗∗^*p* < 0.01, ^∗^*p* < 0.001.*

**Table 6:** Direct and indirect effects and 95% confidence intervals in final model 3.

Model pathways	Estimated effect	95% CI	
		Lower bounds	Upper bounds
**Total effects**			
JD→CWB	0.74***	0.568	0.904
JD→JA	0.67**	0.488	0.826
**Direct effects**			
JD→PD	–0.51***	–0.644	–0.385
JD→CWB	0.37***	0.211	0.529
JD→JA	0.58***	0.368	0.785
PD→JA	–0.18*	–0.337	–0.022
PD→CWB	–0.25**	–0.422	–0.072
JA→CWB	0.36***	0.054	0.666
**Indirect effects**			
JD→PD→CWB	0.13*	0.030	0.228
JD→PD→JA	0.09*	0.021	0.160
JD→PD→JA→CWB	0.03*	0.022	0.038
JD→JA→CWB	0.21*	0.029	0.389

*JD, job demands; PD, psychological detachment; JA, job anxiety; CWB, counterproductive work behavior. ^∗^*p* < 0.05, ^∗∗^*p* < 0.01, *^∗^p* < 0.001.*

## Discussion

Based on the stressor-detachment model, we confirmed that psychological detachment and job anxiety partially and sequentially mediate the relation between job demands and CWBs. Further, psychological detachment mediates the relation between job demands and job anxiety. Compared with the original stressor-detachment model, two additional path coefficients are significant (i.e., job demands to CWBs and psychological detachment to CWBs) in our model. This might be explained by CWBs working as both performance and strain reactors (Fida et al., 2015). Our research thereby makes several contributions to the stressor-detachment model and CWB literature.

### Theoretical Implications

Our research has some theoretical implications. Primarily, this study extends the stressor-detachment model’s application by demonstrating that psychological detachment mediates job demands’ respective relations with job anxiety and CWBs. Previous research focused mostly on detachment’s moderating effect, with the mediating effect largely neglected (Sonnentag, 2011; Germeys and De Gieter, 2016). Thus, our study helps deepen understanding of the stressor-detachment model, and responds to Sonnentag’s (2011) call to test the mediating effect of psychological detachment. The present study also added subsequent performance (i.e., CWBs) to the stressor-detachment model. The study moved beyond the limitations of prior research, which has mainly considered the relationships among stressors, psychological detachment, and strain (Safstrom and Hartig, 2013; Pereira and Elfering, 2014). Through empirical study, we found that job demands (a form of stressor) and job anxiety (a form of strain) have positive effects on CWBs (a form of performance), while psychological detachment has a negative effect on CWBs. These results are similar to those of DeArmond et al. (2014), and are also broadly in line with the stressor-detachment model.

Second, our study extended the existing research on CWBs. Previous research has explained the formation mechanism of CWBs based mainly on the stressor-emotion model: the stressor elicits negative feelings and employees are prone to engage in CWB under their influence (Spector and Fox, 2005a). Though some research has examined the relationships among job demands, job-related affect (e.g., job anxiety), and CWBs (Spector and Fox, 2002; Balducci et al., 2011), few studies have examined the relation between psychological detachment and CWBs. This study reveals that CWBs can be explained by job demands and its relationship with psychological detachment and job anxiety. Thus, we proposed a new perspective to explain the formation mechanism of CWBs by introducing it into the stressor-detachment model, thereby opening a new avenue for further research.

Third, we tested the role of job anxiety in the stressor-detachment model. There is only limited research in which job anxiety, as a specific strain reaction, is incorporated in the stressor-detachment model. Through the literature review, we identified that earlier research tends to take fatigue as a strain reaction, but few consider job anxiety. The current study’s results suggest that job demands and psychological detachment act as antecedent variables and CWBs as an outcome variable of job anxiety. Our study shifts from the previous studies’ focus on personality traits (antecedent variables) (Gramstad et al., 2013; Starrenburg et al., 2013) and turnover intention (outcome variable) (Jensen et al., 2013; Vanderpool and Way, 2013; Husain et al., 2016). Thus, this study enriches understanding of the antecedent and outcome variables of job anxiety and contributes to existing knowledge about job anxiety by incorporating it as a key part of the stressor-detachment model.

### Practical Implications

Our results also have several important practical implications. First, they confirm that job demands are positively related to job anxiety and CWBs, and negatively correlated with psychological detachment. These relationships should be considered by organizations in seeking to reduce job demands. For instance, job design and performance assessment should be based on the practical working capability of employees, and each should be allocated reasonable tasks. Thereby, staff could finish work on time and avoid continuing to work in off-job time. If the job demands cannot be reduced, organizations could create more opportunities for employees to attend training, for example, in setting priorities, time-management skills, and job skills, to help them to work more efficiently (Sonnentag et al., 2014) and avoid perceiving a high workload and time pressure. According to Rothbard et al. (2005), managing the boundaries between work and family depend on each employee’s preference: an employee that wishes to integrate the two may set blurring role boundaries, while one who prefers to segment may set clear role boundaries. Therefore, to diminish work-family conflict, employees must be permitted to set boundaries between work and family in accordance with their own preference. At the same time, a more segmenting policy such as flextime should be implemented as this would not be detrimental to integrators and may marginally help segmentors (Rothbard et al., 2005).

Given that psychological detachment influences job anxiety and CWBs, employees should pursue leisure activities during off-job time (Sonnentag, 2012), such as exercising or developing new interests (Fritz et al., 2010a). Moreover, before starting work each day, it is helpful for employees to create a list (in order of priority) of work tasks that need to be completed that day (Fritz et al., 2010b): workers can, thereby, complete tasks through a planned approach, avoiding the need to engage in work-related thoughts or activities during off-work time. In addition, debiasing trainings are the useful strategies to help employees to set priority (Ceschi et al., 2017). Further, both mangers and co-workers should not be available for and should avoid work-related communication with employees during off-work time.

Finally, to avoid negative effects from job anxiety, organizations should select and train employees carefully. For example, the 16 Personality Factor Questionnaire should be used to assess the personality characteristic of anxiety when recruiting (Chakravarthy and Chandramohan, 2015). In addition, organizations can seek to eliminate employees’ worries or fear of failing to achieve organizational goals by setting realistic objectives for employees. Organizations could also provide training courses to teach employees’ practical skills to cope with job anxiety, or create more opportunities for employees to receive psychological counseling and consultation.

### Limitations and Future Research

Our study has several limitations that should be overcome in future research. Primarily, our study employed a cross-sectional design. Therefore, it would be premature to draw exact conclusions about causality. Although some studies have validated the causal linkage between job anxiety and CWBs (Salami, 2010; Muschalla and Linden, 2014), alternative explanations are difficult to rule out. For instance, in our research, we assumed that employees with high job anxiety tend to engage in CWBs. Another possibility is that employees are punished for engaging in CWBs, which increases their job anxiety. For example, if an employee breaks the organization’s rules, they may be punished, for example, through a pay deduction. Thereafter, they may worry about another pay deduction if they break the rules again, hence increasing their job anxiety. This may explain why the path coefficient between CWBs and job demands was significant (β = 0.66, *p* < 0.001) in the reverse model. Thus, to confirm causal links, longitudinal and experimental studies should be employed in future research.

Second, the research data were collected through a self-report form and all variables were assessed based on employees’ perceptions, which may lead to CMB (Proenca et al., 2017). We took some measures to control the effect of CMB during questionnaire design and data analysis. However, Spector (2006) suggested that CMB may be not a serious issue in practice (Prottas et al., 2017). Further, the CFA marker technique demonstrated that CMB was not serious. Nonetheless, CMB is a potential limitation. Therefore, some other measurement methods, such as coworker assessment, leader assessment, depth interview, and behavioral observation should be employed in future research.

Third, as our data were only collected from three large, state-owned coal mines in northern China, the generalizability of our findings to other organizations is questionable. There are huge differences in the job characteristics and environment between coal mines and other industries (Qing-gui et al., 2012). Thus, to enhance the universality of the current conclusions future research should test our model in more diverse industries.

## Ethics Statement

This study was carried out in accordance with the recommendations of ethics committee of China University of Mining and Technology with written informed consent from all subjects. All subjects gave written informed consent in accordance with the Declaration of Helsinki. The protocol was approved by the ethics committee of China University of Mining and Technology.

## Author Contributions

YC designed and drafted the work. SL collected the data. QX revised the manuscript. CH analyzed data for the study.

## Conflict of Interest Statement

The authors declare that the research was conducted in the absence of any commercial or financial relationships that could be construed as a potential conflict of interest.
